# SRC-3/TRAF4 facilitates ovarian cancer development by activating the PI3K/AKT signaling pathway

**DOI:** 10.1007/s12032-022-01944-0

**Published:** 2023-01-10

**Authors:** Ying Wang, Xia Luo, Nayiyuan Wu, Qianjin Liao, Jing Wang

**Affiliations:** Hunan Cancer Hospital and the Affiliated Cancer Hospital of Xiangya School of Medicine, Central South University, Changsha, China

**Keywords:** SRC-3, Ovarian cancer, Stemness, Migration, Invasion

## Abstract

**Objective:**

Ovarian cancer is the seventh most common cancer in women, and it causes many deaths in women worldwide. Patients with ovarian cancer have a poor prognosis and low survival rate. This study aimed to explore the role of the SRC-3/TRAF4/PI3K/AKT pathway in ovarian cancer development.

**Methods:**

SRC-3 and TRAF4 expression in ovarian cancer cell lines were assessed using qRT-PCR and western-blotting. The expression of SRC-3 and TRAF4 in ovarian cancer cells was downregulated by transient transfection with sh-RNAs. An MTT assay was performed to evaluate cell proliferation. Cell migration and invasion were measured using a Transwell assay. Cell stemness was detected using a cell spheroidization assay and western blotting. The expression levels of stem cell factors and PI3K/AKT pathway proteins were determined by qRT-PCR and western blot analysis.

**Results:**

SRC-3 and TRAF4 were upregulated in ovarian cancer cell lines. TRAF4 is a downstream factor of SRC-3, and the protein level of TRAF4 was regulated by SRC-3. SRC-3 knockdown reduced TRAF4 expression. Silencing SRC-3 or TRAF4 inhibited cell proliferation, migration, and invasion, as well as the expression of stem cell factors. Furthermore, sh-TRAF4 as well as treatment with LY294002, the PI3K/Akt inhibitor, inhibited the phosphorylation of Akt and PI3K, thus repressing the activation of PI3K/AKT signaling pathway in ovarian cancer cell lines. However, TRAF4 overexpression reversed the effect of SRC-3 silencing on cell proliferation, migration, invasion, and stemness.

**Conclusion:**

Our study demonstrated that SRC-3/TRAF4 promotes ovarian cancer cell growth, migration, invasion, and stemness by activating the PI3K/AKT pathway.

**Supplementary Information:**

The online version contains supplementary material available at 10.1007/s12032-022-01944-0.

## Introduction

Ovarian cancer is the seventh most common cancer and is one the leading cause of malignancy-related mortality in women [1]. Most ovarian cancers are derived from serous tubal intraepithelial carcinoma, while others develop from serous endometrial intraepithelial carcinoma [2]. Owing to the lack of obvious symptoms and effective detection methods, 70 percent of ovarian cancers are diagnosed at stage III or IV with extensive metastases in the abdominal cavity [3, 4]. Debulking surgery and chemotherapy are regarded as the most common treatment options for ovarian cancer [2, 5]. According to ovarian cancer surgery guidelines (ESGO 2017), surgical resection of macroscopic tumors is the first choice for patients in their early stages [6]. The chemotherapeutic modality, platinum/taxane treatment, for instance, would be administered to the patients [7]. However, for patients with advanced stages of tumors (III and IV), neoadjuvant chemotherapy is administered before surgery. Bevacizumab and paclitaxel are the first-line treatment options [7]. However, the risk of recurrence for advanced ovarian cancer is very high (~ 70%) [2, 8–10]. Chemotherapy is the principal treatment for recurrent ovarian cancer, and the combination of platinum and other drugs will be useful [7, 11]. However, once ovarian cancer relapses, the tumors develop rapidly with therapy resistance to some extent [2]. It causes many deaths in women worldwide, and patients with ovarian cancer have a poor prognosis and low survival rate [12].

Previous studies have shown that ovarian cancer stem cell transformation could be one of the reasons for ovarian cancer development [13]. Cancer stem cells then give rise to chemoresistant recurrent tumors at the metastatic sites. It has been reported that ovarian cancer stem cells are related to survival after conventional chemotherapy [7]. Numerous studies have been conducted to elucidate the biological mechanisms that regulate ovarian cancer stemness. In regard to ovarian cancer, various specific markers of stemness, such as Nanog, SOX2, and OCT4, have been used to isolate and characterize ovarian cancer stem cells. Moreover, these stem cell factors have been found to regulate ovarian cancer development [14–16]. PBX1, a stem cell reprogramming factor, mediates chemotherapy resistance in ovarian carcinomas [17]. The Wnt signaling pathway is involved in ovarian cancer development by regulating the balance between stemness and differentiation of ovarian cancer stem cells [18]. However, the underlying mechanisms of cancer stem cells in ovarian cancer development remain unclear, and a deeper understanding of cancer cell migration, invasion, and their interaction with stem cell factors is needed to facilitate studies on ovarian cancer prevention and the development of novel therapeutic approaches.

Steroid receptor coactivator 3 (SRC-3) is an oncogenic nuclear receptor coactivator belonging to the p160 family of coactivators [19]. Numerous studies have shown that SRC-3 is overexpressed in ovarian carcinomas and other human malignant diseases [19–21]. SRC-3 has been regarded as a marker of aggressive diseases because it plays an important role in drug resistance and activation of multiple pathways, such as E2F and AKT signaling [21–23]. In addition, SRC-3 directly inhibits the function of the tumor suppressor p53 to exert its oncogenic role [24]. Increased SRC-3 expression in ovarian cancer predicts chemoresistance and poor prognosis [21]. SRC-3 interacts with nuclear receptors such as estrogen receptor (ER) to enhance target gene transcription and promote cancer cell proliferation [19, 25]. It has been reported that SRC-3 can control metastasis of ovarian cancer cells [19]. Downregulation of SRC-3 expression in ovarian cancer cells significantly inhibits their migration and spread [21]. However, the mechanism by which SRC-3 is involved in ovarian cancer development remains unclear.

TRAF4 [Tumor necrosis factor (TNF) receptor-associated factor 4], a member of the TRAF protein family, is a critical downstream player of SRC-3 [24, 26]. TRAF4 is expressed at basal levels in normal tissues and cells [26]. However, it is abundantly expressed in various human malignancies [27]. Although it shares a common TRAF domain at the C-terminus with other TRAF family members, unlike other TRAF family members, TRAF4 does not interact with TNF and interleukin receptors [26]. High TRAF4 expression has been detected in different cancers, such as hepatocellular carcinoma, endometrial cancer, and breast cancer [28–30]. Emerging evidence indicates that TRAF4 plays an essential role as an oncogene in cancer development [31]. TRAF4 modulates cell proliferation, apoptosis, migration, and invasion in breast cancer and other cancer types [32, 33]. Previous studies have shown that breast cancer patients with high TRAF4 expression have poor prognosis [34]. Furthermore, overexpression of TRAF4 eliminates the inhibition of cancer cell migration via DR6 knockdown [35]. Liu et al. demonstrated that TRAF4 facilitates HCC cell migration and invasion by activating the PI3K/Akt signaling pathway [28]. Furthermore, it has been reported that *Oct4* is a downstream gene of the PI3K/AKT pathway. TRAF4 increased the expression of *Oct4* in endometrial cancer cells. TRAF4 promotes cell proliferation and migration by activating the PI3K/AKT/Oct4 pathway, which regulates the progression of endometrial cancer [29].

As a downstream factor of SRC-3, the protein level of TRAF4 is regulated by SRC-3 in cells and breast tumors. With the help of AP-1 transcription factor, SRC-3 directly regulates TRAF4 transcription [24]. The expression of TRAF4 is positively correlated with SRC-3 expression in breast tumors [24]. Breast cancer cells with high levels of TRAF4 are more resistant to nitric oxide-induced cell death, similar to cells with high levels of SRC-3 [36]. A previous study reported that TRAF4 was upregulated in endometrial cancer tissues [29]. TRAF4 promotes the development of endometrial cancer by activating the PI3K/AKT pathway and enhancing the phosphorylation of AKT and PI3K [29]. However, it is unclear whether SRC-3 employs a similar TRAF4/PI3K/AKT pathway to facilitate ovarian cancer development.

In this study, we tested the growth, migration, invasion, and stemness of ovarian cancer cells by knocking down expression of SRC-3 or TRAF4. In addition, we verified that the specific binding of SRC-3 and TRAF4 in ovarian cancer cells promoted the development of ovarian cancer by activating the PI3K/AKT pathway. A better understanding of the molecular mechanisms of the SRC-3/TRAF4 axis in the development of ovarian cancer will improve our understanding of carcinogenesis and facilitate additional therapeutic avenues for targeting ovarian cancers.

## Materials and methods

### Plasmid constructs

cDNAs encoding the full-length *TRAF4* was amplified from ovarian cancer cell via reverse transcription-PCR and cloned into pcDNA3.1 (Invitrogen). Short-hairpin RNA (shRNA) targeting *SRC-3* and *TRAF4* were constructed by annealing two pairs of primers. The sense stranded oligos and their respective anti-sense stranded oligos were annealed and cloned into pLKO.1 vector (Invitrogen). A negative control was constructed by the same method with another pair of primers. All constructs were verified by DNA sequencing.

### Cell cultures and transfection

The Anglne cells were obtained from Procell (Wuhan, China). The Other human ovarian cancer cell lines and normal ovarian epithelial cells were obtained from American Type Culture Collection (ATCC, USA) and were maintained in Dulbecco's modified Eagle’s medium (DMEM; Gibco, USA) containing 10% fetal bovine serum (FBS) (Gibco, USA) and 1% penicillin–streptomycin solution (Gibco, USA) at 37 °C.

CAOV-3 and SKOV3 cells were seeded at a density of 1 × 10^6^ cells/well in 6-well plates and transfected with sh-SRC-3 and sh-TRAF4 alone or together with pcTRAF4 using Lipofectamine 2000 reagent (Invitrogen) in serum-free OPTI-MEM (Invitrogen), according to the manufacturer's instructions. CAOV-3 and SKOV3 cells were incubated in a fresh medium containing 20 μM LY294002. The cells were harvested at specific time points, and protein expression was analyzed.

### MTT assay

Cell proliferation was determined using the 3-(4, 5-dimethylthiazol-2-yl)-2, 5-diphenyltetrazolium bromide (MTT) colorimetric assay (Sigma Aldrich) according to the manufacturer's instructions. Cells transfected with sh-SRC-3 or sh-TRAF4 alone, or together with pc-TRAF4, were seeded at a density of 1 × 10^4^ cells/well in 96-well plates. After 1, 2, and 3 d, the cells were washed twice with PBS. MTT (5 mg/mL) was added into the cells and incubated with the cells at 37 °C. After 3 h, 100 μL of DMSO was added to dissolve the formazan crystals. The density of each well was measured at 570 nm wavelength using a microplate reader (Bio-Rad Laboratories, USA).

### Transwell assay for migration

Transwell migration assays were performed using 8.0-μm pore inserts (BD Biosciences, USA). Cells (5 × 10^4^) were suspended in 200 μL of DMEM and loaded into the upper wells, and 600 μL of DMEM with 5% FBS was added to the lower chambers. Cells were incubated at 37 °C for 2 d. The cells were then fixed with 4% paraformaldehyde and stained with 0.1% crystal violet solution for 4 h. The number of migratory cells was determined by counting five random areas of constant size per well.

### Transwell assay for invasion

Transwell invasion analysis was performed using 100 μL Matrigel (1:5)-coated inserts (BD Biosciences, USA). Then, 5 × 10^4^ cells were added to each insert. DMEM containing 5% FBS was added to the lower chamber. The cells and medium were incubated at 37 °C for 2 d. After incubation, the medium was removed and the cells were washed twice with PBS. The cells were then fixed with 4% paraformaldehyde and stained with 0.1% crystal violet solution for 4 h, and the number of invading cells was confirmed by counting five random areas of constant size per well.

### qRT-PCR analysis

Total RNA was extracted from cells using TRIzol reagent (Invitrogen, USA), according to the manufacturer's instructions. cDNA was obtained by reverse transcription using a reverse transcription kit (M1701; Promega, USA). The expression of *SRC-3, TRAF4, CD44, CD133, SOX2, OCT3/4*, and *NANOG* was detected by qRT-PCR using the SYBR Green Master Mix (Takara). *GAPDH* mRNA was used as an internal control and relative expression changes were calculated using the 2^−ΔΔCt^ method.

### Western blot

The proteins used for western blotting were extracted from the tissues and cells using RIPA lysis buffer. Five microliters of the lysates were removed for protein concentration measurement (Bio-Rad Protein Assay Dye Reagent Concentrate). Total protein (30 μg) was subjected to sodium dodecyl sulfate–polyacrylamide gel electrophoresis. Proteins were then transferred to nitrocellulose membrane filters (Millipore, USA). After blocking with 5% milk in PBST at room temperature for 30 min, the membranes were incubated with primary antibodies at room temperature for 60 min. The primary antibodies used in this study were as follows: anti-SRC-3 (mouse mAb #2115, 1:1000), anti-CD44 (mouse mAb #3570, 1:1000), anti-CD133 (rabbit mAb #86781, 1:1000), anti-SOX2 (mouse mAb #4900, 1:1000), anti-NANOG(mouse mAb #4893, 1:1000), anti-TRAF4 (rabbit mAb #18527, 1:1000), anti-p-AKT (rabbit mAb #4058, 1:1000), anti-AKT (rabbit mAb #75692, 1:1000), anti-p-PI3K (rabbit mAb #17366, 1:1000), and anti-PI3K (antibody #4255, 1:1000) were purchased from Cell Signaling Technology; anti-GAPDH (mouse mAb, AC002, 1:5000) and anti-OCT3/4 (rabbit pAb, A7920, 1:1000) were purchased from ABclonal. After washing three times with PBST buffer, the membranes were incubated with HRP-conjugated secondary antibodies (Sigma-Aldrich) for 1 h at room temperature. Images were visualized using a Licor Odyssey imager (LiCor Inc., Lincoln, NE, USA). The relative density of the bands was analyzed and quantified using Image Quant software from the Licor Odyssey imager.

### Colony formation assay

About 200 cells were inoculated in a low-adhesive 6-well plate containing stem cell culture medium (DMEM/F12 + 20 ng/mL EGF, 10 ng/mL βFGF, and 10 μL/mL B27) (Gibco, USA) and incubated at 37 °C without serum. After two weeks, an inverted microscope was used to observe clones with diameters greater than 50 μm.

### Statistical analysis

All assays were repeated at least three times to ensure accuracy. The results of multiple experiments are presented as mean ± SD. Statistical analyses were performed using the GraphPad software (version 5.0; GraphPad Software, USA). *P*-values were calculated using one-way analysis of variance (ANOVA). A *P*-value of < 0.05 was considered statistically significant.

## Results

### *SRC-3* and *TRAF4* were upregulated in ovarian cancer cells

**Fig. 1:**
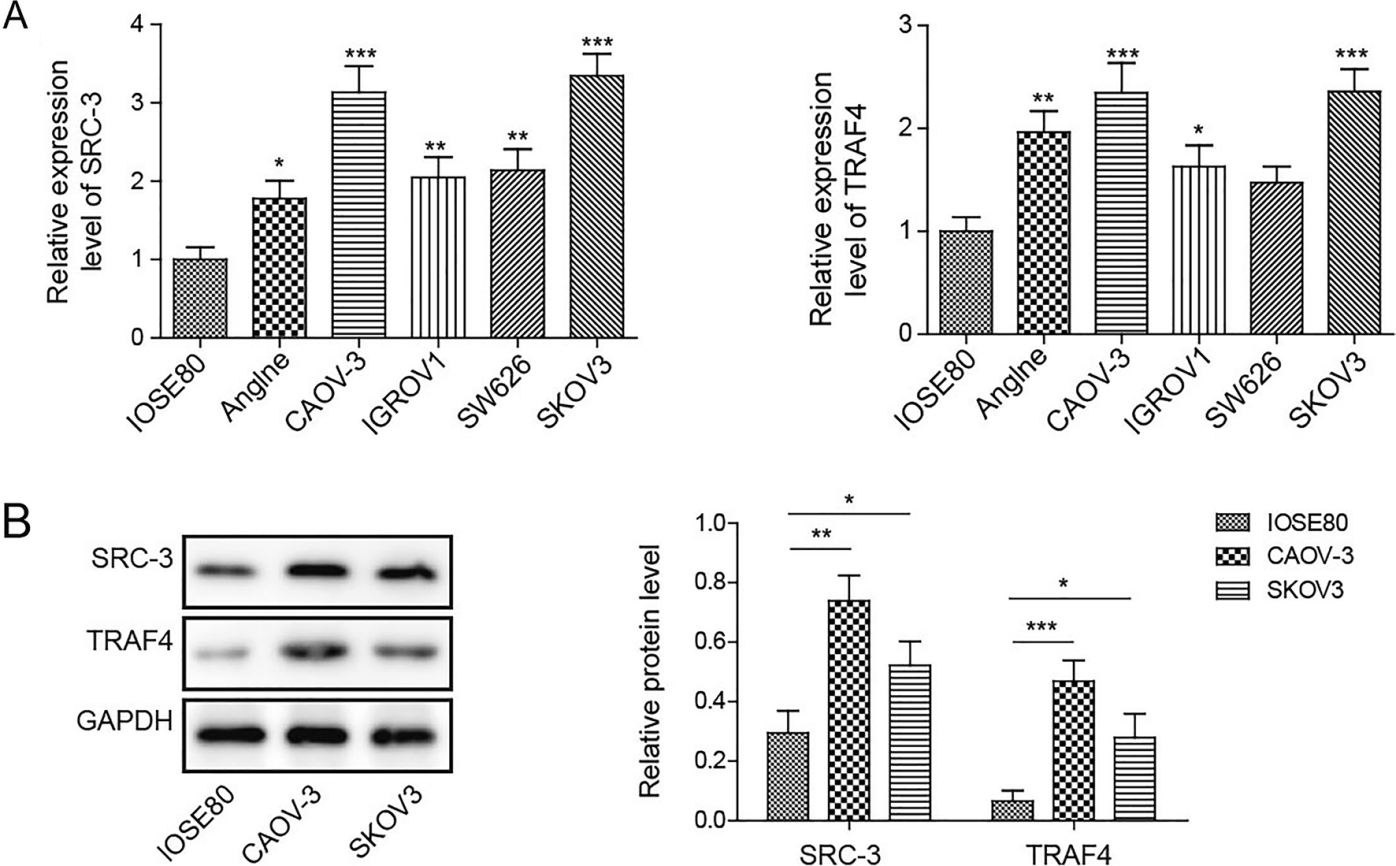
SRC-3 and TRAF4 were upregulated in ovarian cancer cells. **A** The mRNA level of SRC-3 and TRAF4 were detected by qRT-PCR in ovarian cancer cells and normal ovarian epithelial cells. **B** The protein levels of SRC-3 and TRAF4 were detected by western blot in CAOV-3 and SKOV3 cells and normal ovarian epithelial cells. *n* = 3. *P < 0.05, **P < 0.01, ***P < 0.001

We assessed the mRNA levels of *SRC-3* and *TRAF4* in multiple ovarian cancer cell lines using qRT-PCR. As shown in Fig. 1A, the mRNA levels of *SRC-3* and *TRAF4* were increased in ovarian cancer cell lines, such as Anglne, CAOV-3, IGROV1, SW626, and SKOV3, compared to normal ovarian epithelial cells, IOSE80, with the mRNA levels of *SRC*3 and *TRAF4* in CAOV3 and SKOV3 cells showing the highest change (Fig. 1A). In addition, SRC3 and TRAF4 protein expression was detected in IOSE80, CAOV3, and SKOV3 cells. Western blotting results showed that the protein expression of SRC3 and TRAF4 was significantly higher in CAOV3 and SKOV3 cells than in IOSE80 cells (Fig. 1B). Because SRC-3 and TRAF4 were more highly expressed in CAOV-3 and SKOV3 cells than in other ovarian cancer cell lines, we speculated that these two cell lines might be most significantly regulated by the expression of SRC3 and TRAF4. Therefore, these two cell lines were selected for subsequent experiments. Taken together, these results strongly support that SRC-3 and TRAF4 are upregulated in ovarian cancer cells.

### Knockdown of *SRC-3* repressed the growth, migration, invasion, and stemness of ovarian cancer cells

**Fig. 2:**
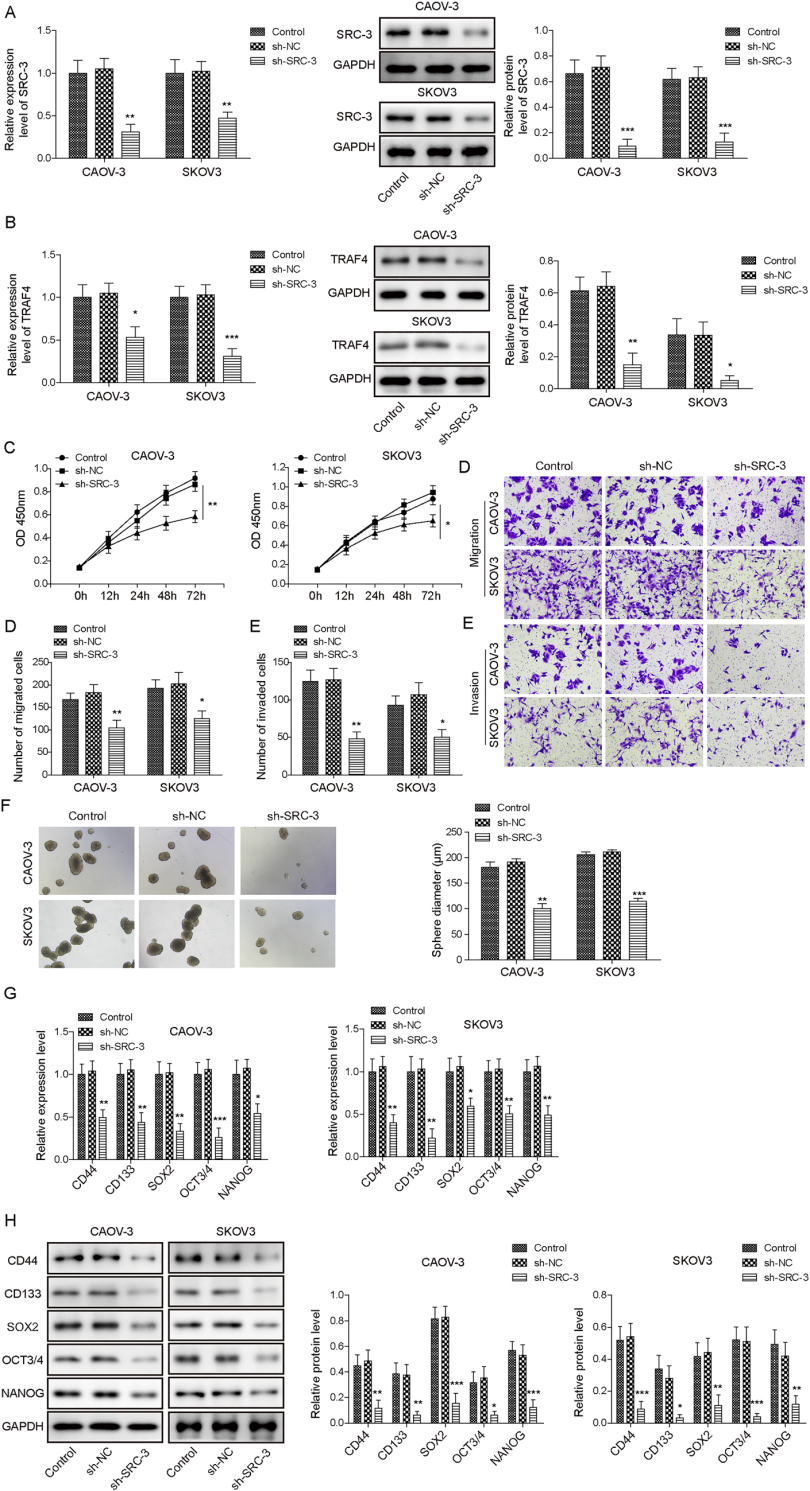
Knocking down SRC-3 repressed the growth, migration, invasion and stemness of ovarian cancer cells. **A** The expression of SRC-3 was decreased in sh-SRC-3 transfected CAOV-3 and SKOV3 cells by qRT-PCR and western blot assay. **B** The expression of TRAF4 was decreased in sh-SRC-3 transfected CAOV-3 and SKOV3 cells by qRT-PCR and western blot assay. **C** Cell proliferation was detected by MTT assay after being transfected with sh-SRC-3. **D** and **E** SRC-3 silence decreased cell migration and invasion by transwell assay. **F** The cell spheroidization test detected the number of CAOV-3 and SKOV3 cells into spheroids after knocking down SRC-3. **G** and **H** The expression of stem cell factors after silencing SRC-3 was analyzed by qRT-PCR and western blot. *n* = 3. *P < 0.05, **P < 0.01, ***P < 0.001

We further investigated the role of SRC-3 in ovarian cancer cell growth, migration, invasion, and stemness. First, we tried to downregulate SRC-3 expression using sh-RNA. The mRNA level of *SRC-3* was dramatically decreased by shRNA-targeting *SRC-3* compared to that in the control and sh-NC groups (Fig. 2A). Furthermore, the protein level of SRC-3 was detected by western blot in *SRC-3* knocked down CAOV-3 and SKOV3 cells. Consistent with our mRNA results, the protein level of SRC-3 was significantly decreased when sh-SRC-3 was transfected into CAOV-3 and SKOV3 cells (Fig. 2A). The expression level of TRAF4 was detected in *SRC-3* knocked down CAOV-3 and SKOV3 cells. qRT-PCR and western blotting results showed that SRC-3 silencing dramatically reduced the mRNA and protein levels of TRAF4 in CAOV-3 and SKOV3 cells (Fig. 2B). Cell proliferation was determined using the MTT assay. As shown in Fig. 2C, SRC-3 silencing significantly decreased the viability of CAOV-3 and SKOV3 cells in a time-dependent manner (Fig. 2C). The migration and invasion of ovarian cancer cells were detected using a transwell assay. Compared to the control group, *SRC-3* knockdown resulted in a significant decrease in cell migration (Fig. 2D). In accordance with the migration results, inhibition of SRC-3 expression substantially decreased the relative invasion of CAOV-3 and SKOV3 cells compared to the control group (Fig. 2E). Furthermore, the effect of SRC-3 silencing on cellular stemness was detected using spheroidization analysis. Similarly, compared to the control group, the sh-*SRC-3* group showed significantly less cell spheroidization (Fig. 2F). Additionally, the expression levels of stem cell factors were detected by qRT-PCR and western blot assays. As shown in Fig. 2G, knockdown of *SRC-3* profoundly decreased the mRNA levels of *CD44, CD133, SOX2, OCT3/4,* and *NANOG* in CAOV-3 and SKOV3 cells compared to the control group (Fig. 2G). Similarly, the protein levels of CD44, CD133, SOX2, OCT3/4, and NANOG dramatically decreased when *SRC-3* was silenced (Fig. 2H). These results suggested that *SRC-3* silencing inhibited the growth, migration, invasion, and stemness of ovarian cancer cells.

### *TRAF4* silencing decreased ovarian cancer cell growth and development

**Fig. 3:**
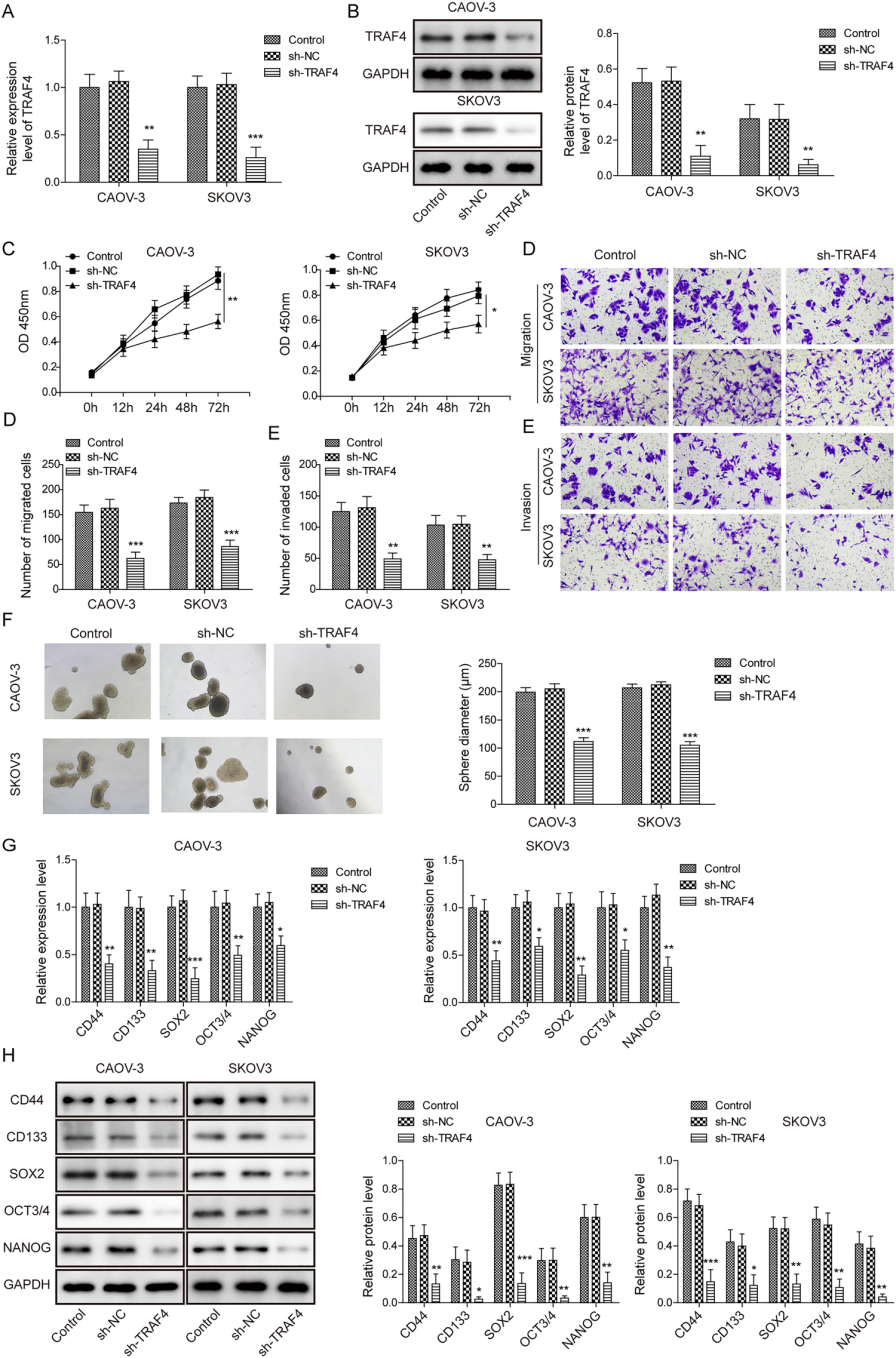
TRAF4 silence decreased ovarian cancer cell growth and development. **A** and **B** The expression of TRAF4 in CAOV-3 and SKOV3 cells after knocking down TRAF4 was analyzed by qRT-PCR and western blot. **C** The effect of silencing TRAF4 on cell proliferation was detected by MTT assay. **D** and **E** Transwell assay detected the effect of TRAF4 knockdown on cell migration and invasion. **F** The cell spheroidization experiment detected the number of cells spheroids when TRAF4 was knocked down. **G** and **H** The expression of knockdown TRAF4 stem cell factor was analyzed by qRT-PCR and western blot. *n* = 3. *P < 0.05, **P < 0.01, ***P < 0.001

Previous studies have suggested that TRAF4 is critical for breast cancer development and acts downstream of SRC-3 [24]. To further explore the role of TRAF4 in ovarian cancer cell growth and development, *TRAF4* was knocked down using shRNA*.* The mRNA and protein expression of TRAF4 was significantly decreased by sh-*TRAF4* (Fig. 3A, B). The MTT assay showed that cell proliferation was sharply decreased when *TRAF4* was silenced by sh-TRAF4 in a time-dependent manner compared with that in the control group (Fig. 3C). Cell migration was suppressed when *TRAF4* was knocked down (Fig. 3D). Similarly, cell invasion was inhibited when *TRAF4* was silenced by shRNA compared to the control group (Fig. 3E). Spheroidization analysis showed that cell spheroidization decreased dramatically when *TRAF4* was silenced (Fig. 3F). In addition, qRT-PCR and western blot assays showed that knockdown of *TRAF4* substantially downregulated the mRNA and protein expression of CD44, CD133, SOX2, OCT3/4, and NANOG in CAOV-3 and SKOV3 cells compared to that in the control group (Fig. 3G, H). These results demonstrated that TRAF4 plays an important role in ovarian cancer cell growth and development.

### SRC-3/TRAF4 promoted ovarian cancer cell growth and development by activating the PI3K/AKT pathway

**Fig. 4:**
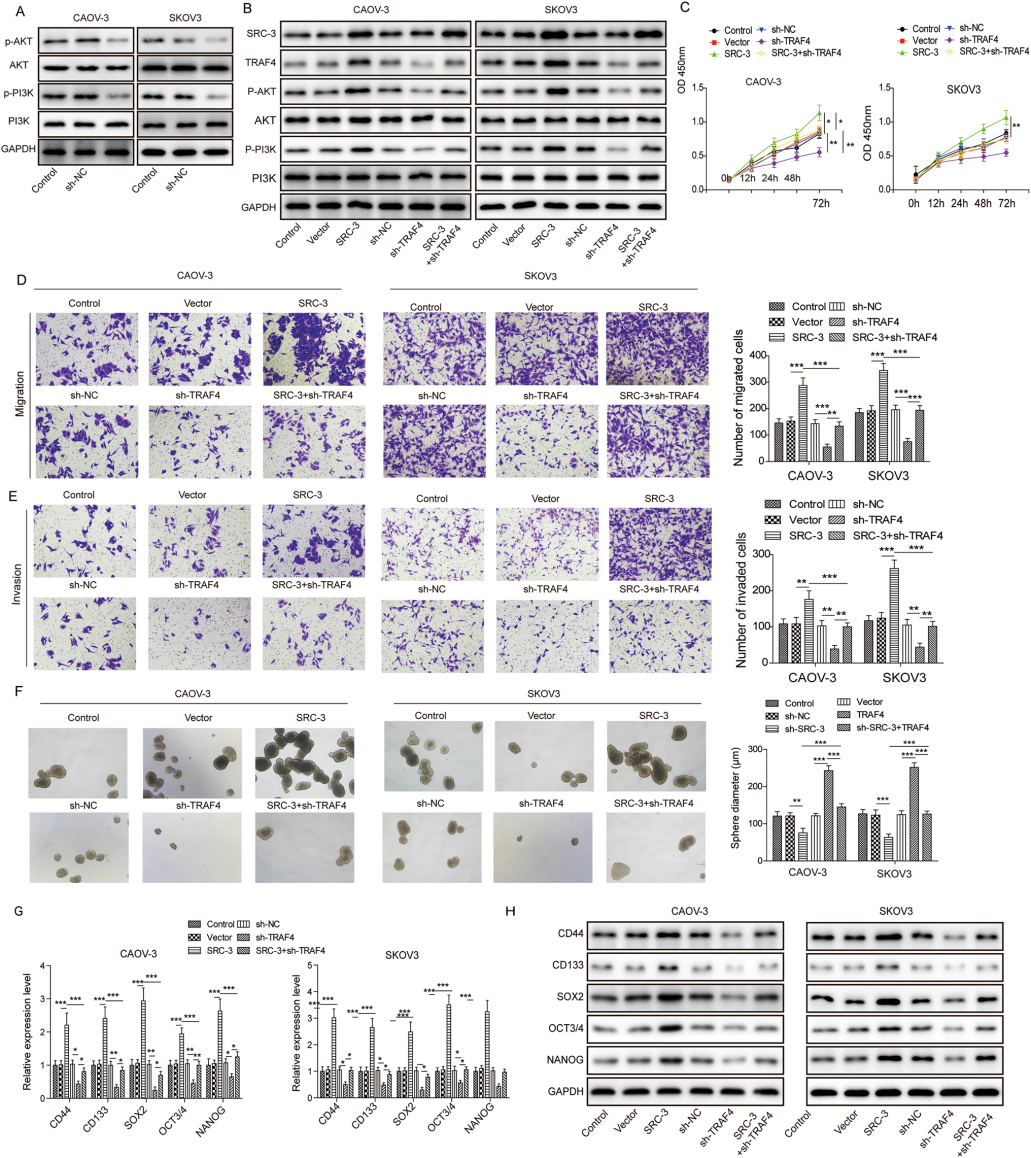
SRC-3/TRAF4 promoted ovarian cancer cell growth and development by activating the PI3K/AKT pathway. **A** Western blotting detected the effect of TRAF4 knockdown on PI3K/AKT signaling pathway in CAOV-3 and SKOV3 cells. **B** Western blot detected the expression of SRC-3, TRAF4, PI3K, AKT, p-AKT and p-PI3K in cells after overexpressing SRC-3 and overexpressing TRAF4. **C** MTT method detected the proliferation of CAOV-3 and SKOV3 cells. **D** and **E** The effect of Transwell analysis on cell migration and invasion. **F** The cell spheroidization test detected the number of spheroids of cells. **G** and **H** The expression of stem cell factors was analyzed by qRT-PCR and western blot. *n* = 3. *P < 0.05, **P < 0.01, ***P < 0.001

Previous studies have reported that TRAF4 activates the PI3K/AKT pathway to promote endometrial cancer development [29]. To further elucidate the mechanism by which TRAF4 promotes ovarian cancer cell growth and development, the expression of p-PI3K/PI3K and p-AKT/AKT was determined by western blotting. As shown in Fig. 4A, the phosphorylation levels of AKT and PI3K dramatically decreased in *TRAF4* silenced CAOV3 and SKOV3 cells (Fig. 4A). LY204002, a specific PI3K/AKT signaling pathway inhibitor, was used to confirm the effect of the PI3K/AKT signaling pathway on the development of ovarian cancer. CAOV-3 and SKOV3 cells were treated with LY204002 to determine phosphorylation levels of PI3K and AKT. Compared with the control group, the phosphorylation levels of AKT and PI3K were significantly downregulated when cells were treated with LY204002 (Supplement Fig. 1A). The MTT assay showed that cell proliferation decreased when cells were treated with LY204002 (Supplement Fig. 1B). Furthermore, the Transwell assay showed that cell migration and invasion were suppressed in cells treated with LY204002 compared to the control group (Supplement Fig. 1C, D). Cell spheroidization was also decreased in LY204002 treated cells (Supplement Fig. 1E). In addition, expression of CD44, CD133, SOX2, OCT3/4, and NANOG was detected. When the cells were treated with LY204002, the mRNA and protein levels of CD44, CD133, SOX2, OCT3/4, and NANOG were significantly decreased, as determined by RT-PCR and western blot assays (Supplement Fig. 1F, G). Taken together, these results suggest that the PI3K/AKT signaling pathway plays a vital role in ovarian cancer development.

To further explore the role of SRC-3/TRAF4 in PI3K/AKT signaling pathway activation, *SRC-3* and sh-*TRAF4* were co-transfected into CAOV-3 and SKOV3 cells. Western blotting was performed to detect the expression of SRC-3, TRAF4, p-PI3K/PI3K, and p-AKT/AKT. Overexpression of SRC-3 upregulated TRAF4, p-AKT, and p-PI3K expression compared with that in the control and vector groups, while it had no effect on AKT and PI3K expression. Transfection with sh-*TRAF4* downregulated TRAF4, p-AKT, and p-PI3K expression, but had no effect on SRC-3, AKT, and PI3K expression compared with that in sh-NC, control, and vector groups. Co-expression of *SRC-3* and sh-*TRAF4* dramatically increased the phosphorylation levels of AKT and PI3K when compared with that in sh-*TRAF4* transfected group (Fig. 4B). Cell proliferation was measured using the MTT assay. The results showed that SRC-3 overexpression significantly increased cell viability and that sh-*TRAF4* had the opposite effect. As expected, co-expression of *SRC-3* and sh-*TRAF4* restored cell viability compared with that in the sh-TRAF4 group (Fig. 4C). A transwell assay was performed to evaluate cell migration and invasion. In accordance with the cell proliferation results, overexpression of SRC-3 dramatically increased cell migration and invasion, while silencing of *TRAF4* inhibited the effect of SRC-3 on migration and invasion (Fig. 4D, E). In addition, spheroidization experiment analysis was used to evaluate cellular stemness. Cell spheroidization was dramatically inhibited when *TRAF4A* was knocked down, whereas it was restored on SRC-3 overexpression (Fig. 4F). Furthermore, the mRNA and protein expression of stem cell factors CD44, CD133, SOX2, OCT3/4, and NANOG were significantly increased in SRC-3 overexpressed cells by qRT-PCR and western blot assays. However, silencing *TRAF4* reversed the effect of SRC-3 on the expression of these proteins (Fig. 4G, H). Overall, our results suggest that SRC-3/TRAF4 activates the PI3K/AKT signaling pathway and plays an important role in the growth, migration, invasion, and stemness of ovarian cancer cells.

### SRC-3 upregulated the expression of TRAF4 to enhance the growth, migration, invasion, and stemness of ovarian cancer cells

**Fig. 5:**
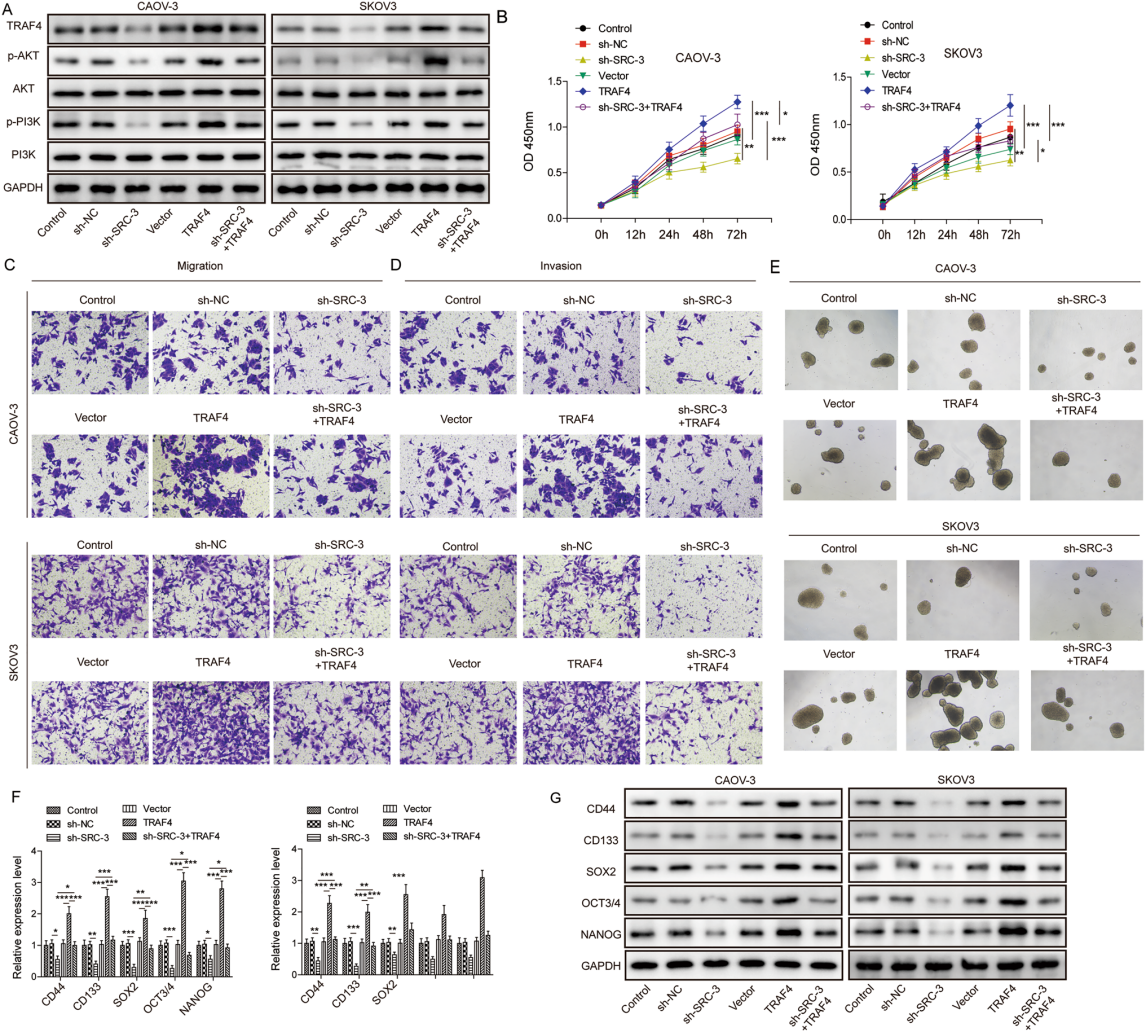
SRC-3 upregulated the expression of TRAF4 to enhance the growth, migration, invasion and stemness of ovarian cancer cells. **A** Western blot detected the expression of SRC-3, TRAF4, PI3K, AKT, p-AKT and p-PI3K in cells after knocking down SRC-3 and overexpressing TRAF4. **B** MTT method detected the proliferation of CAOV-3 and SKOV3 cells. **C** and **D** The effect of Transwell analysis on cell migration and invasion. **E** The cell spheroidization test detected the number of spheroids of cells. **F** and **G** The expression of stem cell factors was analyzed by qRT-PCR and western blot. *n* = 3. *P < 0.05, **P < 0.01, *** P < 0.001

Our previous results demonstrated that SRC-3 could increase the expression of TRAF4 and that TRAF4 could activate the PI3K/AKT pathway. We further investigated the role of the SRC-3/TRAF4/PI3K/AKT pathway in ovarian cancer development. In CAOV-3 and SKOV3 cells, sh-*SRC-3* decreased the expression of TRAF4 as well as the phosphorylation levels of AKT and PI3K, whereas TRAF4 overexpression exerted the opposite effect and increased the expression of TRAF4. Furthermore, overexpression of TRAF4 in cells transfected with sh-*SRC-3* rescued the phosphorylation levels of AKT and PI3K to some extent (Fig. 5A). Similarly, in the MTT assay, sh-*SRC-3* significantly decreased cell viability, whereas TRAF4 overexpression exerted the opposite effect. After knocking down *SRC-3* and overexpressing TRAF4 in the cells, cell viability was reversed compared to *SRC-3* knockdown alone. (Fig. 5B). In addition, cell migration and invasion were detected using a Transwell assay. Consistent with the results reported above, sh-*SRC-3* dramatically inhibited cell migration and invasion, while the overexpression of TRAF4 reversed the inhibitory effect of knockdown of *SRC-3* on migration (Fig. 5C) and invasion (Fig. 5D). Cellular stemness was evaluated using spheroidization analysis. Cell spheroidization recovered when TRAF4 was expressed in *SRC-3* silenced cells (Fig. 5E). The expression of stem cell factors was determined using qRT-PCR and western blot assays. *SRC-3* knockdown substantially downregulated the mRNA and protein expression of CD44, CD133, SOX2, OCT3/4, and NANOG. However, overexpression of TRAF4 restored the expression of these proteins (Fig. 5F, G). These results showed that SRC-3 upregulated the expression of TRAF4 to enhance the growth, migration, invasion, and stemness of ovarian cancer cells.

## Discussion

Over the past decade, increasing evidence has suggested that the stemness of cancer stem cells (CSCs) plays a crucial role not only in the development and progression of malignant diseases but also in the transformation of normal cells into tumor cells [13]. The stemness of CSCs provides them with the principal properties of self-renewal, clonal tumor initiation capacity, and clonal long-term repopulation potential [37]. Previous studies have confirmed that SRC-3 modulates tumorigenesis in several cancers [19–21]. However, the mechanism by which SRC-3 induces this effect remains unclear.

Yi et al. demonstrated that TRAF4 is a downstream gene of SRC-3, and is involved in cell resistance to cytotoxic stress [24]. TRAF4 is upregulated in various cancers and plays a vital role in cancer development and drug resistance [28–30]. Therefore, extensive efforts have been made to uncover the mechanisms by which TRAF4 regulates cell proliferation, apoptosis, migration, and invasion in various cancer cells. TRAF4 was reported to promote cell migration and invasion by activating the PI3K/Akt signaling pathway in hepatocellular carcinoma [28]. Overexpression of TRAF4 can modify the phosphorylation of Akt as well as the expression of Slug, E-cadherin, and vimentin in hepatocellular carcinoma cells [28]. TRAF4 competed with p53 to interact with deubiquitinase HAUSP, and then induced p53 proteasomal degradation and resistance to cytotoxic agents in [38]. In our study, we found that SRC3 can regulate the expression of TRAF4. It may be give us a hint that SRC3 can induce resistance to cytotoxic agent. Moreover, in primary endometrial cancer cells, the stem cell factor Oct4 has been shown to be a downstream target of PI3K/AKT signaling and is involved in endometrial cancer development. The expression of Oct4 was positively regulated by TRAF4. The TRAF4/PI3K/AKT/Oct4 pathway regulates the progression of endometrial cancer by promoting cell proliferation and migration [29]. In our study, the TRAF4/PI3K/AKT pathway was found to be involved in ovarian cancer development. These findings may reflect the important role of the TRAF4/PI3K/AKT pathway in the development of different cancers. However, to our knowledge, this is the first study to show that SRC-3 activates the TRAF4/PI3K/AKT pathway to promote ovarian cancer development.

SRC-3, an oncogenic nuclear receptor coactivator, is overexpressed in human malignant diseases, driving tumor initiation, cell proliferation, and metastasis [19–21]. This induces drug resistance in cancer cells [19]. It also activates multiple pathways to facilitate the development of various cancers [21–23]. It was reported that SRC-3 supported the cancer stem-like cell state of tumor-initiating cells and induced an epithelial-to-mesenchymal (EMT) transition by driving the expression of the master EMT regulators and stem cell markers [39]. The activation of insulin-like growth factor (IGF)/AKT was involved in the SRC-3 on the cell growth and invasiveness in esophageal squamous cell carcinoma cell lines [23]. PFKFB4, a Warburg pathway enzyme, regulates transcriptional reprogramming by activating SRC-3 to drive breast cancer [40]. These findings suggest that SRC-3 is important for breast, prostate, and lung cancer development [19–21]. In contrast, TRAF4 interacts with phosphoinositides (PIPs) to drive breast cancer [41]. TRAF4 activates the AKT signaling cascade to enhance osteosarcoma cell proliferation and invasion [42]. In addition, TRAF4 activates TGF-β receptor signaling to accelerate breast cancer metastasis [43]. The expression of SRC-3 and TRAF4 is closely related [24]. In agreement with previous studies, our results showed that overexpression of TRAF4 neutralized the effect of SRC-3 knockdown and rescued ovarian cancer cell migration and invasion. Collectively, our study demonstrates the involvement of SRC-3/TRAF4 pathway in ovarian cancer development.

Multidisciplinary approaches have been used to treat ovarian cancer, and the most common treatments for ovarian cancer are debulking surgery and chemotherapy [44, 45]. Several chemotherapeutic agents have been approved for ovarian cancer treatment. Platinum-containing drugs (cisplatin and carboplatin) and the taxane family (paclitaxel and docetaxel) are common drugs used for chemotherapy [46]. Carboplatin is a good choice because of its low toxicity and less side effects [47]. Gemcitabine, doxorubicin, and bevacizumab may be the preferred choice [48–50]. The selection of chemotherapeutic agents depends on the stage of ovarian cancer [45]. However, the relapse rates of ovarian cancer are high, and relapse may increase the resistance to chemotherapy drugs [2, 8–10]. Currently, immunotherapies are considered for treating ovarian cancer, even though the success rate is very low [45]. Currently, no FDA approval for immunotherapy is available for ovarian cancer. The scientific community was motivated by encouraging results in other closely related tumor types [45]. As the SRC-3/TRAF4/PI3K/AKT pathway plays a vital role in ovarian cancer development, the SRC-3/TRAF4 pathway may be a promising therapeutic target for ovarian cancer treatment.

## Supplementary Information

Below is the link to the electronic supplementary material.

Supplementary file1 (TIF 3577 kb)—**Supplementary Fig. 1 **SRC-3/TRAF4 promoted ovarian cancer cell growth and development by activating the PI3K/AKT pathway. **A** The effect of LY294002 on the phosphorylation of AKT and PI3K was detected by western blot. **B** After LY294002 treatment, the proliferation of CAOV-3 and SKOV3 cells was detected by MTT method. **C** and **D** The cell migration and invasion after treatment with LY294002 were measured by transwell. **E** Detected the number of CAOV-3 and SKOV3 cells into spheroids after LY294002 treatment by cell spheronization test. **F** and **G** The expression of stem cell factors in LY294002-treated cells was analyzed by qRT-PCR and western blot. *n*=3. *P < 0.05, **P < 0.01, ***P < 0.001

## Data Availability

All data generated or analyzed during this study are included in this article. The datasets used and/or analyzed during the current study are available from the corresponding author on reasonable request.
