# Supplementary figures and images for: SRC-3/TRAF4 facilitates ovarian cancer development by activating the PI3K/AKT signaling pathway
Source: Med Oncol. 2023 Jan 10;40(2):76. doi: 10.1007/s12032-022-01944-0 (PMC9831961; doi:10.1007/s12032-022-01944-0)

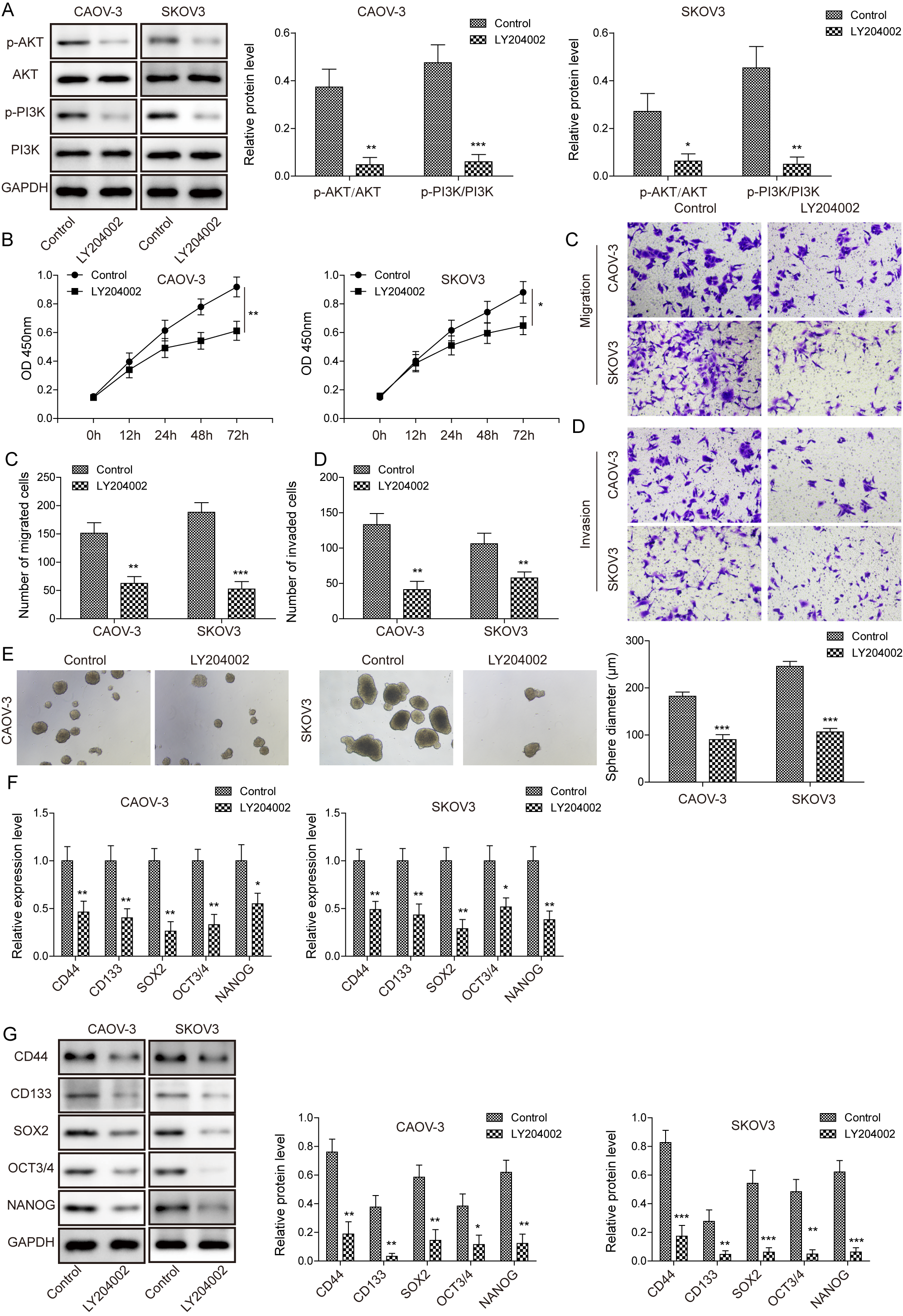

Supplement: Supplementary file 1 — Supplementary file1 (TIF 3577 kb)—Supplementary Fig. 1 SRC-3/TRAF4 promoted ovarian cancer cell growth and development by activating the PI3K/AKT pathway. A The effect of LY294002 on the phosphorylation of AKT and PI3K was detected by western blot. B After LY294002 treatment, the proliferation of CAOV-3 and SKOV3 cells was detected by MTT method. C and D The cell migration and invasion after treatment with LY294002 were measured by transwell. E Detected the number of CAOV-3 and SKOV3 cells into spheroids after LY294002 treatment by cell spheronization test. F and G The expression of stem cell factors in LY294002-treated cells was analyzed by qRT-PCR and western blot. n=3. *P < 0.05, **P < 0.01, ***P < 0.001 [file 12032_2022_1944_MOESM1_ESM.tif]
